# Potential of *Punica granatum* biochar to adsorb Cu(II) in soil

**DOI:** 10.1038/s41598-019-46983-2

**Published:** 2019-07-31

**Authors:** Qinying Cao, Zhihong Huang, Shuguang Liu, Yiping Wu

**Affiliations:** 1 Faculty of Life Science and Technology, Central South University of Forestry and Technology, Changsha, 410004 Hunan China; 2 National Engineering Laboratory for Applied Technology of Forestry and Ecology in South China, Central South University of Forestry and Technology, Changsha, 410004 Hunan China; 3 Department of Earth and Environmental Science, Xi’an Jiaotong University, Xi’an, 710049 Shaanxi China

**Keywords:** Environmental biotechnology, Environmental sciences

## Abstract

Biochar as a promising adsorbent to remove heavy metals has attracted much attention globally. One of the potential adsorbents is biochar derived from *punica granatum* peels, a growing but often wasted resource in tropical countries. However, the immobilization capacity of *punica granatum* peel biochar is not known. This study investigated the physicochemical properties of *punica granatum* peel boichars pyrolyzed at 300 °C and 600 °C (referred as BC300 and BC600), and the efficiency and mechanisms of Cu(II) adsorption of five types of material treatments: BC300, BC600, soil only, and soils with biochar amendment BC300 and BC600, respectively, at the rate of 1% of the soil by weight. The results show that BC300 had higher yield, volatile matter content and organic carbon content, and larger pore diameter, but less ash content, surface area, pH, and cation exchange capacity than BC600. The Cu(II) adsorption capacity onto biochars and soils with biochar were greatly influenced by initial ion concentration and contact time. The Cu(II) adsorption capacity of biochar, independent of pyrolysis temperature, was around 52 mg g^−1^. The adsorption capacity of the soil amended with biochar nearly doubled (29.85 mg g^−1^) compared to that of the original soil (14.99 mg g^−1^), indicating superb synergetic adsorption capacity of the biochar-amended soils. The adsorption isotherms showed monolayer adsorption of Cu(II) on biochar, and co-existence of monolayer and multilayer adsorption in soils with or without biochar amendment. Results also suggest that the adsorption process is spontaneous and endothermic, and the rate-limiting phase of the sorption process is primarily chemical. This study demonstrates *punica granatum* peel biochar has a great potential as an adsorbent for Cu(II) removal in soil.

## Introduction

Copper (Cu(II)) is one of the heavy metals widely used in industrial manufacture^1^. Anthropogenic activities, such as mining and smelting, electroplating, petroleum refining and brass manufacture, are the main sources of Cu(II)^2^. Although Cu(II) is one of the essential micro-nutrients needed by living organisms^3,4^, the excessive doses of Cu(II) can cause serious problems to humans such as anaemia, hypoglycemia, stomach intestinal distress, and even kidney damage and eventual death^5,6^. Therefore, it is necessary to develop effective methods to remove Cu(II) from polluted water and soil. Recently, removal of Cu(II) from wastewater via adsorption is a promising technology with easy operation, high efficiency and relatively low-cost and insensitivity to toxic substances^1^, which has been adopted widely by water treatment plants^3,7,8^.

Biochars are produced through the pyrolysis of agricultural and forest residues with limited or no oxygen^9^. Biochar is widely used as an adsorbent in removing heavy metal ions of aqueous solution in the recent years^10^, which is due to its large surface area, high porosity and pH, and a large number of active functional groups such as hydroxy, carboxy, carbonyl^11,12^. Adsorption capacities are highly correlated with the properties of the biochars^1^. The properties of biochars are mainly determined by the feedstock material and the pyrolysis conditions (e.g. pyrolysis temperature)^13,14^. Several materials like plant residues, animal manures, industrial wastes and sewage sludge have been investigated as potential feedstock for biochar production^15–18^.

It is well known that China is one of the largest agricultural countries in the world. More than 260,000 tons of *punica granatum* are produced annually in China^19^. Most of the *punica granatum* residues are discarded, wasting a large amount of potential biomass resources as well as causing pollution to the environment. Therefore, it is necessary to find an effective way to deal with this problem. Using *punica granatum* peel as feedstock to produce biochar could be a feasible way to make bioenergy production with a low-cost, environment-friendly, and sustainable management of the *punica granatum* peel waste. However, so far no studies have been conducted on the production and application of biochar derived from *punica granatum* peels.

The overall aim of this work was to evaluate *punica granatum* peel biochar as a potential adsorbent to immobilize Cu(II) in contaminated soils. The objectives were to (1) investigate the effect of pyrolysis temperatures (300 °C and 600 °C, respectively) on the physicochemical properties of *punica granatum* peel biochar; (2) evaluate the influences of initial concentration and contact time on the sorption efficiency of Cu(II) onto adsorbents (i.e., BC300, BC600, soil, soil with BC300, and soil with BC600, respectively); and (3) understand the Cu(II) adsorption mechanisms (i.e., isotherms, kinetics, and thermodynamics) of these adsorbents.

## Results and Discussion

### The physicochemical and morphological characterization of biochars

In this study, the yield of biochar decreases from 46.6% to 28.0% as the temperature increases from 300 °C to 600 °C (Table 1). The decrease of biochar yield with tempeature is similar to the reports by Selvanathan *et al*.^20^ and Dai *et al*.^21^, which is attributed to the loss of volatiles and the condensation of aliphatic compounds because of increasing temperature^22^. The high yield of biochar is often considered as an important factor in practical application. At the same pyrolysis temperature (300 °C and 600 °C), *punica granatum* peels had a higher yield of biochar compared to that from orange peels (yield as 37.2% at 300 °C, 26.7% at 600 °C)^23^ and sugarcane bagasse (yield as 26.1% at 300 °C, 12.0% at 600 °C)^24^.

**Table 1:** The physicochemical properties of *pomegranate* peel biochars pyrolyzed at 300 °C and 600 °C.

Biochar	Yield (%)	Ash content (%)	Volatile matter (%)	Surface area (m^2^ g^−1^)	Pore diameter (nm)	pH(1:2.5)	CEC (cmol kg^−1^)	Organic carbon (%)
BC300	46.6	18.8	21.3	41.28	17.08	7.71	53.20	54.8
BC600	28.0	39.4	6.7	195.32	3.34	10.76	74.11	46.7

Notes: BC300 and BC600, *pomegranate* peel biochar pyrolyzed at 300 °C and 600 °C, respectively. CEC stands for the cation exchange capacity.

It is also observed that the volatile matter content decreases from 21.3% to 6.7% when the pyrolysis temperature increases from 300 °C to 600 °C (Table 1) as the thermal degradation of biochar is gradually complete with the increase of pyrolysis temperature. In addition, ash content in BC600 is higher than that in BC300. In this work, *punica granatum* peel biochar has a higher volatile and ash content compared to rambutan peel biochar^20^ at the same pyrolysis temperature. Our results are consistent with the negative correlation between volatile and ash content^25–27^. These phenomena could be attributed to the volatilization of abundant inorganic components^28^.

In this work, biochar produced at the low-temperature (BC300) has a higher organic carbon content compared to that at the high-temperature (BC600) (54.8% *vs*. 46.7%) (Table 1). Our results are similar to reports that the organic carbon content decreased with the increasing pyrolysis temperature^25,29^, indicating that the enhancement of aromatization increases with the increasing temperature^27^. In this study, organic carbon content of *punica granatum* peel biochar is from 1.6 to 2.2 times higher than that of sugarcane bagass biochars^24^ at the same pyrolysis temperature. This may be related to the properties of the biomass materials.

**Figure 1:**
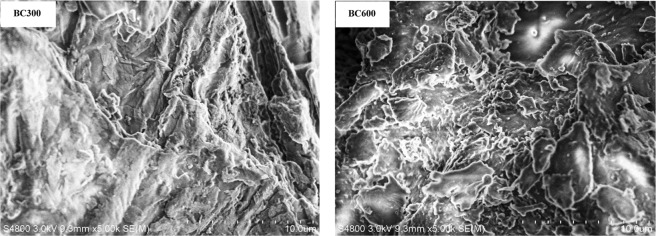
SEM images of *pomegranate* peel biochars at 30 kev; magnification 500. Notes: BC300 and BC600, pomegranate peel biochar pyrolyzed at 300 °C and 600 °C, respectively.

Surface area of biochar increased from 41.28 to 195.32 m^2^ g^−1^ as temperature increases from 300 °C to 600 °C (Table 1). The increase of surface area of biochar with pyrolysis temperature have also been reported in the literature^3,30,31^. However, the increase in surface area of biochar showed a wave increase with increasing pyrolysis temperatures^32–34^. This phenomenon was related with destruction of both ester groups and aliphatic alkyl, and the exposure of aromatic lignin core as increasing pyrolysis temperature^25^. The pore diameter of BC600 (3.34 nm) is less than that of BC300 (17.08 nm), which is less than that of poultry manure biochars^30^ at the same pyrolysis temperature (300 °C and 600 °C). The main reason for this difference still remains to be further investigated. Additionally, our results are consistent with the opinion that there is a positive correlation between surface area and micropore volume^35^, and the pore size distribution is a key factor responsible for an increase in surface area in biochar^36^. Together with the biochar yield, the total surface area of biochar was estimated to be 18.86 and 54.60 m^2^ g^−1^ biomass for BC300 and BC600, respectively. Apparently, the surface area yield of BC600 was more than two times surface area of BC300. In addition, the SEM images showed that the surface morphology of biochars is featured by the numerous mesopores with varying sizes and shapes (Fig. 1). Compared with BC300, pores on BC600 were well developed, and the pore distribution on BC600 was relatively dense. Hence, it can be concluded that the surface structural changes in biochar are significantly influenced by pyrolysis temperature.

As shown in Table 1, pH increased significantly from 7.71 to 10.76 with the increasing pyrolysis temperature. Similar results have been reported by other researchers^21,26,29,37^. In the literatures, the range of pH was from 3.16^33^ to 12.10^38^ varied with pyrolysis temperature from 60 °C to 800 °C, with a mean value of 8.66. This phenomenon may be related with the release of the acidic surface groups during the pyrolysis process^39^. The alkaline pH of biochar has a liming effect on acidic soils, thereby probably increasing plant productivity^39^. High-ash biomass generates biochars with slightly greater CEC and charge density upon normalization of CEC to surface area^26^. In this study, CEC increased from 53.20 to 74.11 cmol kg^−1^ as pyrolysis temperature increase from 300 °C to 600 °C (Table 1). Komkiene *et al*.^40^ found an increase in the CEC of silver birch biochars from 5.09 cmol kg^−1^ to 5.71 cmol kg^−1^ with pyrolosis temperature from 450 °C to 700 °C, indicating the creation of the functional groups of hydroxyl and carboxylic acid in the oxidation process^41,42^. In contrast to our results, the CEC of some biochars decreased with increasing pyrolysis temperatures^39,43^, which could be due to the reduction of carbonylic and carboxylic functional groups^44^.

Elemental compositions and molar ratios of biochars have been extensively used to analyze the effects of pyrolysis temperature on the functional chemistry of biochars^24^. Table 2 shows the elemental composition and molar ratios of biochars pyrolyzed at 300 °C and 600 °C. Our results showed that the higher pyrolysis temperatures resulted in the biochar with higher carbon content, lower contents of hydrogen, oxygen and nitrogen (Table 2). This feature was in agreement with findings of previous studies^32,44,45^. The increase in carbon content with temperature may be resulted from enhancement of carbonization^46^, while the lower content of hydrogen, oxygen and nitrogen at high temperatures could have been attributed to the breaking of weaker bonds in biochar structure together with the loss of water, —OH, —C=O, —COOH and hydrocarbons during the carbonization process^47^. The H/C ratios decreased from 0.09 to 0.03, indicating the formation of structures containing saturated carbons such as aromatic rings^48^. The O/C and (O + N)/C ratios decreased with the increasing pyrolysis temperature, which is reflective of the reduction of oxygen-containing polar functional groups on biochar surface^34,49^.

**Table 2:** The elemental composition of biochars pyrolyzed at 300 °C and 600 °C.

Biochar	C (%)	H (%)	O (%)	N (%)	O/C	H/C	(O + N)/C
BC300	46.64	4.06	39.31	1.34	0.84	0.87	0.87
BC600	62.34	1.82	36.11	0.25	0.58	0.02	0.58

Notes: BC300 and BC600, *pomegranate* peel biochar pyrolyzed at 300 °C and 600 °C, respectively.

### Chemical characterization of different soil treatments

The chemical properties of three soil treatments are presented in Table 3. Compared with the control soil, the soils amended with the addition of 1% biochar (BC300 and BC600, respectively) have higher pH, CEC, and organic carbon content (Table 3). In contrast with soil with BC300, soil with BC600 result in greater changes in pH and CEC. An increase in soil pH has been reported with the application of biochar^50,51^. Increase of soil pH is due to the alkaline pH of biochar which is attributed to the presence of negatively charged carboxyl, hydroxyl and phenolic groups on biochar surfaces^52^. Increased soil pH with biochars contributes to the CEC increase by reducing the leaching of base cations in competition with H^+^ ions via enhanced binding to negatively charged functional sites of biochar^53,54^. Biochar-induced change in the physical and chemical properties of soil can further influence the sorption of metal ions^26,49^.

**Table 3:** The chemical properties of three soil treatments.

Treatment	pH	CEC (cmol kg^−1^)	Organic carbon (g kg^−1^)	Cu(II) (mg kg^−1^)
soil	4.34	21.33	23.56	0.03
soil with BC300	4.47	38.20	37.62	0.01
soil with BC600	5.31	54.11	29.88	—

Notes: soil, control treatment; soil with BC300 and soil with BC600, soil amended with addition of BC300 and BC600, respectively in mass ratio of 1%. CEC stands for the cation exchange capacity.

### Effect of initial metal ions concentration and adsorption isotherms

**Figure 2:**
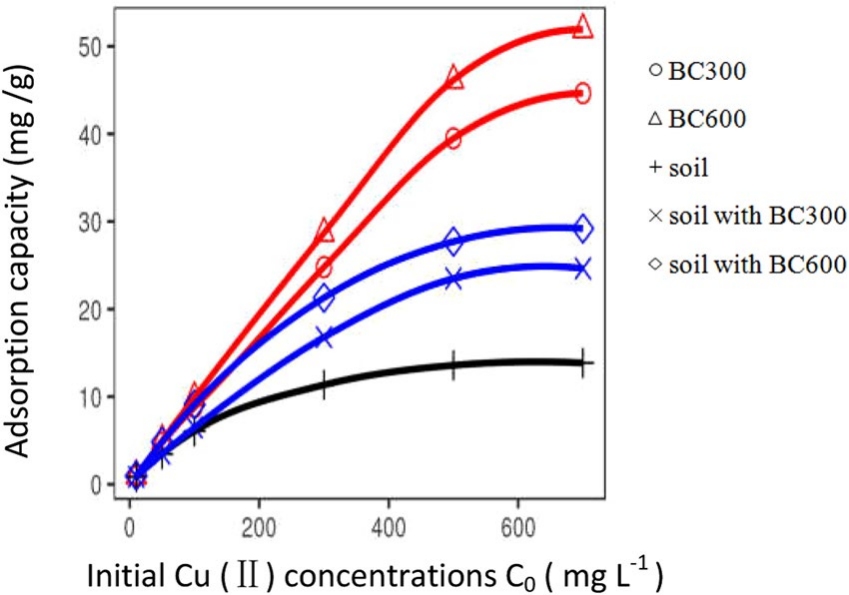
Effect of initial Cu(II) concentration on adsorption capacity of Cu(II) onto different adsorbents (adsorbent dosage = 0.5 g, initial Cu(II) concentration = 10, 50, 100, 300, 500, 700 mg L^−1^, initial solution pH = 5.0 ± 0.1, contact time = 25 h, temperature = 25 °C).

Current results are in agreement with the observation that the adsorption capacity of adsorbents has a close correlation with the initial concentration of metal ions in the reaction system^55^. As shown in Fig. 2, the adsorption efficiency of Cu(II) onto different adsorbents (soil, soil with BC300, soil with BC600, BC300, and BC600) increased with increasing initial Cu(II) concentrations. However, the equilibrium Cu(II) concentrations, where the adsorption rates start to level off, were different among adsorbents. The BC600 and BC300 has the highest equilibrium Cu(II) concentrations of about 500 mg L^−1^. In contrast, the equilibrium Cu(II) concentration of soil with BC300 and soil with BC600 was about 300 mg L^−1^, and that of the control soil was about 200 mg L^−1^. The adsorption capacity is attributed to the presence of active sites on the adsorbent surface^7^. There are greater available active sites with faster metal adsorption during the initial stage (i.e., at low Cu(II) concentration), whereas a few active sites are available with stable adsorption at the equilibrium stage^7^.

Moreover, we found that the adsorption efficiency of Cu(II) onto adsorbents increases with increasing initial concentrations of Cu(II) (Fig. 2) and follows the order of BC600 (51.92 mg g^−1^) > BC300 (44.63 mg g^−1^) > soil with BC600 (29.19 mg g^−1^) > soil with BC300 (24.63 mg g^−1^) > soil (13.85 mg g^−1^) at the Cu(II) concentration of 700 mg L^−1^. Biochars pyrolyzed at the high-temperature have higher adsorption capacities of heavy metals compared with those pyrolyzed at low-temperatures^40,56,57^, which is due to biochar properties such as high pH, CEC, and surface area^58^. In contrast to our results, Li *et al*.^29^ found that Cd(II) adsorption capacities of water hyacinth derived biochars decreased with increasing pyrolysis temperature because biochars produced at low pyrolysis temperatures have numerous oxygen-containing functional groups that serve as effective binding sites for metal ions via complexation^59,60^. More interestingly, the adsorption capacity of Cu(II) onto soil amended with biochar doubled compared to the control soil in this study. Also, Feng *et al*.^61^ reported that the soils with bagasse biochar addition increased adsorption capacities. It is highly likely that biochar with higher CEC and negatively charged surface could enhance the electrostatic adsorption of Cu(II) in soil^62^. On the other hand, it is attributed to rich oxygen-containing functional groups such as carboxylic and phenolic hydroxyl on the surface of biochar that can form stable surface complex with Cu(II)^60^.

**Figure 3:**
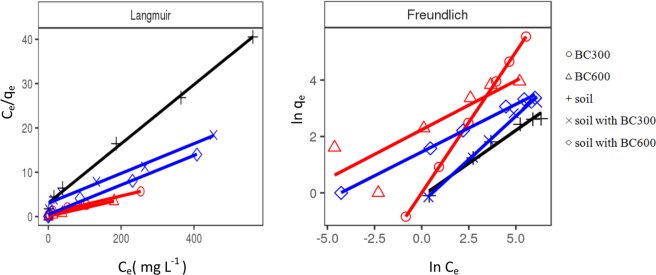
Plots for Langmuir and Freundlich isotherms for adsorption of Cu(II) onto different adsorbents (The legends of “Freundlich” are the same with that of “Langmuir”).

Langmuir and Freundlich models are useful to evaluate the distribution of metal ions between the aqueous and solid phases^57,63^ and the maximum adsorption capacities of adsorbent^62^. Figure 3 shows plots of Langmuir and Freundlich isotherms for adsorption of Cu(II) onto different adsorbents. According to the value of correlation coefficient (R^2^) in Table 4, the Langmuir isotherm model describes well the adsorption of Cu(II) onto both BC300 and BC600 (R^2^ > 0.98), which indicates that the monolayer adsorption occurs on homogeneous surfaces with no interactions among adsorbed metal ions^64^. Our results are consistent with those obtained by Ali *et al*.^27^. Furthermore, Komkiene *et al*.^40^ reported that biochars derived from scots pine and silver birch for the removal of Cu(II) fitted well with the Freundlich adsorption isotherms. The differences in absorption characteristics of biochars could be explained by different feedstocks^65^. In contrast to BC300 and BC600, the Cu(II) adsorption onto soil, soil with BC300, and soil with BC600 can well fit with both of the two models with the coefficients higher than 0.98, which suggested both the monolayer adsorption and the multilayer adsorption on adsorbent surfaces^27,66^. Furthermore, the prediction values of maximum adsorption capacity (q_m_) (Table 4) can well explain the experimental data (see Fig. 3). Compared with the Cu(II) adsorption capacities of other adsorbents reported in literature, the BC300 (51.02 mg g^−1^) and BC600 (53.19 mg g^−1^) exhibited quite a good adsorption performance (see Table 5). The value of 1/n is between 0 and 1 (Table 4), indicating that the adsorption process of Cu(II) onto adsorbents was favorable under the studied experimental conditions^67^. These results proved that BC300 and BC600 could be used as a potential sorbent for the removal of Cu(II) from contaminated soil.

**Table 4:** Langmuir and Freundlich isotherm parameters for the adsorption of Cu(II) onto different adsorbents.

Adsorbent	Langmuir model			Freundlich model		
	q_m_ (mg g^−1^)	K_L_ (L mg^−1^)	R^2^	K_F_ (mg g^−1^)	1/n	R^2^
BC300	51.02	0.03	0.98	0.80	0.79	0.92
BC600	53.19	0.18	1.00	0.79	0.79	0.92
soil	14.99	0.02	1.00	0.95	0.45	0.98
soil with BC300	29.85	0.01	0.98	0.70	0.61	0.99
soil with BC600	30.03	0.07	0.99	0.23	0.34	1.00

**Table 5:** Comparison of the maximum monolayer adsorption of Cu(II) ions on various low-cost adsorbents.

Type of Biomass	Pyrolysis temperature	Absorption condition		q_m_	Reference
	(°C)	pH	Temperature (°C)	(mg g^−1^)	
*pomegranate* peel biochar	300	5.0	25	51.02	this study
*pomegranate* peel biochar	600	5.0	25	53.19	this study
Poplar sawdust	—	4.0	25	3.24	Sciban *et al*.^88^
Coconut tree sawdust	—	6.0	25	3.89	Putra *et al*.^89^
Canola straw biochar	400	5.0	25	0.59	Tong *et al*.^3^
Soybean straw biochar	400	5.0	25	0.83	Tong *et al*.^3^
Peanut straw biochar	400	5.0	25	1.40	Tong *et al*.^3^
Hardwood biochar	300	6.2	25	4.21	Liu *et al*.^70^
Pine wood biochar	700	6.2	25	4.46	Liu *et al*.^70^
Corn straw biochar	600	5.0	25	12.52	Chen *et al*.^90^
Hardwood biochar	450	5.0	22	6.79	Chen *et al*.^90^
Hardwood biochar	500	4.8	20	7.44	Han *et al*.^91^
Coir fibre	—	5.5	30	9.43	Shukla *et al*.^92^
Jute fibres	—	5.0	35	4.23	Shukla *et al*.^93^
Cotton fibre	—	5.0	25	6.12	Paulino *et al*.^94^
Rice husks biochar	300	5.0	24	6.26	Pellera *et al*.^6^
Dried olive pomace biochar	300	5.0	24	7.07	Pellera *et al*.^6^
Silver birch	—	4.0	30	0.13	Bojarczuk *et al*.^95^
Switch grass biochar	500	4.8	20	7.12	Han *et al*.^91^
Compost biochar	300	5.0	24	10.14	Pellera *et al*.^6^
Eggshell	—	6.0	25	34.48	Putra *et al*.^89^
Orange waste	—	5.0	24	10.26	Pellera *et al*.^6^
Tea waste	—	5.0–6.0	25	48.00	Amarasinghe *et al*.^96^
Aquatic plant	—	5.0–6.0	25	10.37	Keskinkan *et al*.^97^
Sugarcane bagasse	—	6.0	25	3.65	Putra *et al*.^89^
Switch grass	—	5.0	25	31.00	Regmi *et al*.^98^
Irish peat moss	—	5.0–6.0	25	17.60	Keskinkan *et al*.^97^
Palm oil fruit shell	—	6.5	20	60.00	Hossain *et al*.^99^
Groundnut shells	—	5.0	60	4.46	Shukla *et al*.^92^
Wheat bran	—	—	20	51.50	Özer *et al*.^100^
Enteromorpha compressa biochar	500	—	25	75.10	Kim *et al*.^101^
Rambutan peels biochar	600	—	25	217.30	Selvanathan *et al*.^20^
Residual biomass	—	4.0	—	28.34	Lezcano *et al*.^102^
Root of rose biochar	450	4.0	30	60.74	Khare *et al*.^103^
Hazelnut shell activated carbon	—	6.0	50	58.27	Demirbas *et al*.^2^
Grape bagasse activated carbon	—	5.0	45	43.47	Demiral *et al*.^104^
Orange peels activated carbon	—	5.0	25	67.32	Romerocano *et al*.^105^
Olive stone activated carbon	—	5.0	30	17.67	Bohli *et al*.^106^

Notes: “–” stands for the data unreported in the literature.

### Effect of contact time and adsorption kinetics

**Figure 4:**
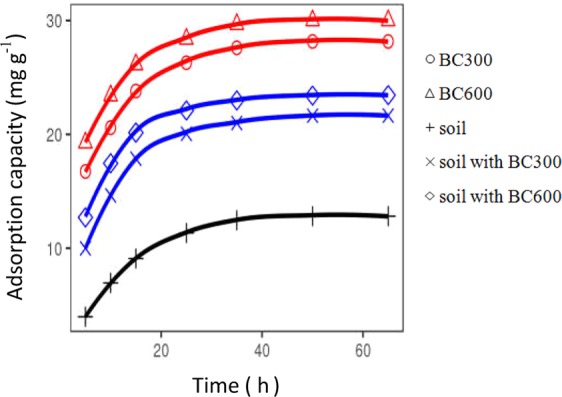
Effects of contact time on adsorption capacity of Cu(II) onto different adsorbents (adsorbent dosage = 0.5 g, contact time = 5, 10, 15, 25, 35, 50, 65 h, initial Cu(II) concentration = 300 mg L^−1^, initial solution pH = 5.0 ± 0.1, temperature = 25 °C).

The relationship between contact time and the adsorption efficiencies of Cu(II) onto different adsorbents is illustrated in Fig. 4. The adsorption rate of Cu(II) onto those adsorbents followed three stages: (1) rapid adsorption during the initial 15 h; (2) slow adsorption lasting from 15 h to 35 h; and (3) equllibrium state starting at the 35^th^ h. Hossain *et al*.^68^ found a large amount of Cu(II) can be bound rapidly onto the adsorbent at the initial stage. Lu *et al*.^69^ demonstrated that slow adsorption was attributed to the remaining vacant active sites that are difficult to be occupied because of the repulsive forces between Cu(II) on the solid and liquid phases. At the same contact time, the adsorption capacity varied with the five adsorbents (Fig. 4) and ranked in the following decreasing order: BC600, BC300, soil with BC600, soil with BC300, and soil. Comparison with control soil, the adsorption capacity increased by 1.7 times for soils with BC300 and 1.8 times for soil with BC600, respectively.

**Figure 5:**
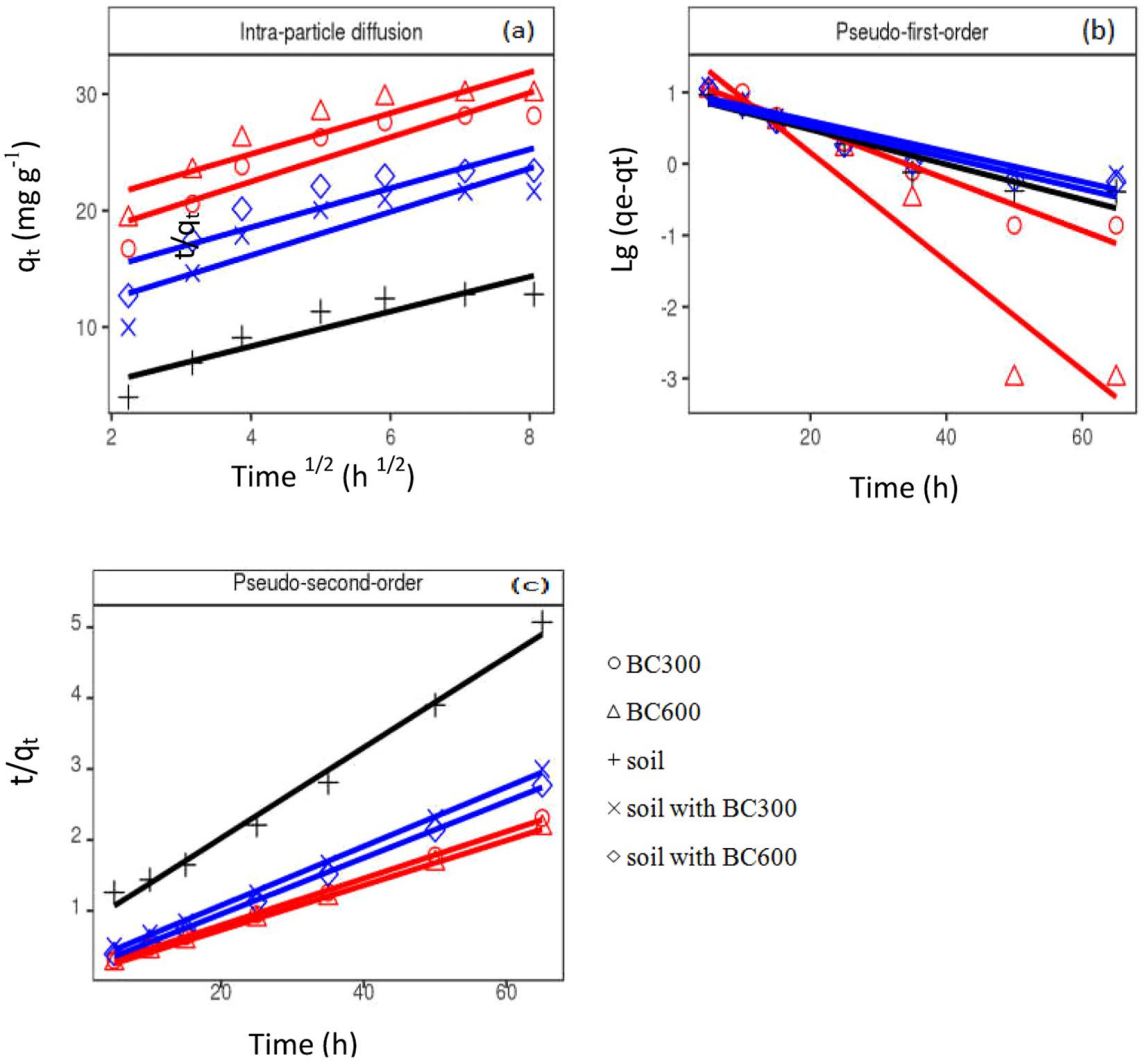
Plots for the intra-particle diffusion, Pseudo-first-order and Pseudo-second-order sorption kinetics of Cu(II) onto different adsorbents.

Kinetic model parameters of Cu(II) adsorption onto the five adsorbents are shown in Table 6. In terms of correlation coefficients (R^2^), the pseudo-second-order kinetic model was in good agreement with the kinetic experimental data (R^2^ > 0.99). A good linear relationship was presented between kinetic experimental data and pseudo-second-order kinetic model (Fig. 5c). In addition, the theoretical equilibrium adsorption capacities (q_e_) calculated using the pseudo-second-order kinetic model were consistent with the equilibrium adsorption capacities (Q_e_) obtained from the contact time study (Table 6). Several other Cu(II) adsorption studies using biochars such as rambutan peel, hardwood and corn stover biochar^20,70^ showed that the adsorption process followed the pseudo-second-order kinetic model. The model assumes that the adsorption process consists of physical adsorption and chemical adsorption^71^, and chemical adsorption is the rate-limiting step^72^.

**Table 6:** Kinetic model parameters for the adsorption of Cu(II) onto different adsorbents.

Adsorbent	Q_e_	Intra-particle diffusion model			Pseudo-first-order model			Pseudo-second-order model		
		*K*_*p*_	C	R^2^	q_e_	K_f_	R^2^	q_e_	Ks	R^2^
	(mg g^−1^)	(g mg^−1^ h^−1/2^)	(mg g^−1^)		(mg g^−1^)	(h^−1^)		(mg g^−1^)	(mg g^−1^ h^−1^)	
BC300	28.30	1.91	14.85	0.84	16.85	0.08	0.96	30.21	0.01	1.00
BC600	30.00	1.75	17.86	0.82	47.74	0.18	0.92	31.75	0.01	1.00
soil	13.22	1.49	2.38	0.85	9.27	0.06	0.92	15.63	0.01	0.99
soil with BC300	22.34	1.86	8.72	0.81	10.65	0.05	0.90	23.92	0.01	1.00
soil with BC600	24.01	1.68	11.87	0.78	9.24	0.05	0.90	25.19	0.01	1.00

To investigate the rate-limiting step of Cu(II) sorption onto the sorbents, the intra-particle diffusion model was employed to fit with the sorption kinetic data. The relationship between q_t_ and t^1/2^ should be linear if intraparticle diffusion is involved in the sorption process. Moreover, if the linear relation passes through the base point, the rate limiting step is mainly controlled by intra-particle diffusion during the adsorption process^73,74^. In this study, the plot of q_t_ and t^1/2^ is a multilinear plot (Fig. 5a), which did not pass through the base point, indicating that the sorption process consists of multiple stages^75^. Hafshejania *et al*.^24^ described the nitrate adsorption by intra-particle diffusion model as the three distinct linear portions–fluid transport, film diffusion, and surface diffusion. The intercepts (C) were nonzero (Table 6), indicating that the sorption processes might involve both the rapid surface sorption and slower intraparticle diffusion through the sorbents occurred simultaneously^73^. In addition, the intercept (C) values provides good information about the boundary layer thickness, that is, the larger intercept means the greater boundary layer effect^76^. The C value of BC600 was larger than the that of BC300 (Table 6). Similar results were reported by Kolodynska *et al*.^73^ who indicated that the biochars obtained at higher temperatures have the more evident boundary layer effect.

### Thermodynamic studies

Thermodynamic analysis of the adsorption process is to investigate whether the process is spontaneous or not. In this study, thermodynamic parameters for the adsorption of Cu(II) onto different adsorbents are listed in Table 7. The values of ΔG° presented here were in the range from −13.26 to −0.13 kJ mol^−1^. As shown in Table 7, the negative values of ΔG° imply that the adsorption processes are thermodynamically spontaneous in nature. The values of ΔG° were within the ranges of −20 to 0 kJ mol^−1^ ^59^, which indicated that adsorption mechanism is dominated by physical adsorption^77^. The values of ΔG° gradually decreased with the increasing temperature (Table 6), which suggests that the higher temperature is more favorable for the adsorption process. The higher temperature provides sufficient energy for heavy metal ions adsorption on the surficial and interior layers of adsorbent^63^. The positive value of ΔS° indicates the increased randomness

**Table 7:** Thermodynamic parameters for the adsorption of Cu(II) onto different adsorbents.

Treatment	Temperature	ΔG°	ΔH°	ΔS°
	(°C)	(kJ mol^−1^)	(kJ mol^−1^)	(J mol^−1^ K^−1^)
	15	−2.37		
BC300	25	−5.97	101.08	359.22
	35	−9.56		
	15	−3.44		
BC600	25	−8.36	138.13	491.56
	35	−13.27		
	15	−0.26		
soil	25	−1.06	22.69	79.70
	35	−1.86		
soil with BC300	15	−0.13		
	25	−1.93	51.69	179.92
	35	−3.73		
soil with BC600	15	−0.45		
	25	−2.67	63.25	222.02
	35	−4.19		

at the solution-solid interface during the adsorption process^53^. The positive ΔH° shows that the adsorption process is endothermic. In this study, the values of ΔH° (Table 7) were within the ranges of 22.69 to 138.13 kJ mol^−1^. Generally, physical adsorption occurs mainly with the ΔH° value of less than 84 kJ mol^−1^, and chemical adsorption dominates with the ΔH° value in the range from 84 to 420 kJ mol^−1^ ^78^. Therefore, the values of ΔH° presented in Table 7 indicated that adsorption of Cu(II) on the biochars (both BC300 and BC600) is dominated by chemical adsorption. In contrast, the adsorption of Cu(II) on the soil and soil with biochars is mainly governed by the physical adsorption.

## Conclusions

The physicochemical properties of *punica granatum* peel biochars (BC300 and BC600) are greatly influenced by the pyrolysis temperature (300 °C and 600 °C, respectively). The Cu(II) removal efficiency and adsorption capacity onto biochars and soils with biochar were controlled by initial ion concentration and contact time. The maximum adsorption capacities of Cu(II) onto soil, soil with BC300, soil with BC600, BC300 and BC600 were 14.99, 29.85, 30.03, 51.02 and 53.19 mg g^−1^, respectively. These results revealed that the application of biochars (BC300 and BC600) can significantly improve the adsorption capacities of the soil for Cu(II). Adsorption characteristics of Cu(II) onto biochars fitted well by the Langmuir model,and adsorption characteristics of Cu(II) onto soil and soil amended with biochars were fitted well by both Langmuir and Freundlich models, indicating that there are monolayer adsorption and multilayer adsorption. Sorption kinetics of Cu(II) onto biochars and soils with biochar can be described by the pseudo-second-order mode. The thermodynamic parameters show that the adsorption is a spontaneous, endothermic, and entropy increasing process. This study indicates biochar derived from *punica granatum* peel is an effective and cheap adsorbent for the removal of Cu(II) in soils.

## Materials and Methods

### Preparation of biochars, soil, and solutions

Fresh red *Punica granatum* peels were collected from the Lintong, Xi’an City, Shan’xi Province, China. They were washed with deionized water, chopped into 1 × 1 cm^2^, and dried in an oven at 105 °C for 24 h. The pyrolysis process was conducted in a furnace (Fisher Scientific, USA) with N_2_ gas at 300 °C and 600 °C separately. The heating rate was set at 15~20 °C/min. The targeted temperatures (300°Cand 600 °C, respectively) are maintained for 2 h before cooling to room temperature. The biochars derived at 300 °C and 600 °C are referred to as BC300 and BC600, respectively. Biochar samples were ground and sieved to achieve the particle size of 0.75~1.00 mm for use in this study.

The testing soil was collected from the Ecological Station of the Central South University of Forestry and Technology (28°08’N, 113°00’E), Changsha City, Hunan Province, China. The soil sampling depth was 5~20 cm. The soil is red with a parent material from Quaternary sediments. The mixed sample soil was air-dried, ground and sieved to achieve the particle size of 1.75~2.00 mm.

A stock solution of 1000 mg L^−1^ Cu(II) was made by dissolving an appropriate amount of CuSO_4_∙5H_2_O in 0.1 mol L^−1^ NaCl solution, which was used as an electrolyte to control the ionic strength of metal ions. Then the stock solution was further diluted in distilled water to get solutions of desired concentrations at 10, 50, 100, 300, 500 and 700 mg L^−1^.

### Characterization of biochars

The pyrolysis yield of biochars was calculated as the ratio of the weight of pyrolysis product to that of the original material. The ash content was calculated by determining the weight loss of 1 g biochar after its combustion in a crucible at 800 °C^79^. Following the same procedure, the volatile matter was determined at 950 °C^80^. The surface area and pore diameter of biochars were measured using the Brunauer-Emmett-Teller (BET) method^81^. The surface physical morphology was studied by a scanning electron microscope (SEM) (S-4800, Tokyo, Japan). The pH was measured using a volumetric ratio of 1: 2.5 (solid: liquid) by a pH meter (PXS-270, Shanghai, China). The cation exchange capacity (CEC) of biochars was determined using 1 mol L^−1^ NH_4_OAc (pH 7.0), and the concentration of exchangeable base cation was measured using an atomic absorption spectrometer (AAS) (PinAAcle 900, PerkinElmer, America). The organic content was obtained by the potassium dichromate oxidation heating method^82^. The elemental composition (C, H, N and O) of biochars was determined by an elemental analyzer (Vario EL, Elementar Analysensysteme GmbH, Germany). Atomic ratios of (O + N)/C and H:C were calculated to evaluate the polarity and aromaticity of biochars.

### Experimental design

We designed five experimental treatments: (1) soil, (2) BC300, (3) BC600, control treatment, (4) soil with BC300, and (5) soil with BC600. For treatments (4) and (5), 1000 g soil samples were weighted and put into the plastic pot (20 cm in top diameter, 12 cm in bottom diameter, and 15 cm in height). Biochar was added to the soil samples in a mass ratio of 1% (BC300 and BC600, respectively) and then mixed evenly. All treatments were repeated four times. For all the treatments, the soil moisture content was adjusted to 70% of the field capacity. After being incubated at 25 °C for 30 days, the treated soil was air-dried and sieved to achieve the particles of 1.75~2.0 mm.

### Adsorption experiments

#### Adsorption isotherms experiments

The Langmuir (Eq. (1)) and Freundlich (Eq. (2)) isotherms are often adopted to model the adsorption process^83^:

1 $$\frac{{C}_{{\rm{e}}}}{{q}_{e}}=\frac{{C}_{e}}{{q}_{m}}+\frac{1}{{K}_{L}{q}_{m}}$$

2 $$\mathrm{ln}\,{q}_{e}=\frac{1}{n}\,\mathrm{ln}\,{C}_{e}+\,\mathrm{ln}\,{K}_{F}$$

where *q*_*e*_ (mg g^−1^) is the adsorbed amount of metal ions at the equilibrium time, *q*_*m*_ (mg g^−1^) is the maximum adsorption amount of metal ions when they form a monolayer on the adsorbent surface, *C*_*e*_ (mg L^−1^) is the metal ions concentration of the equilibrium aqueous phase, *K*_*L*_ (L mg^−1^) is the Langmuir equilibrium constant, relating to the adsorption capacity and rate. *q*_*m*_ and *K*_*L*_ are evaluated by the intercept and slope of the plot of *C*_*e*_*/q*_*e*_ against *C*_*e*_. *K*_*F*_ (mg g^−1^) is the Freundlich constant, relating to the adsorption capacity, and 1*/n* is the intensity of the adsorbent. *K*_*F*_ and *1/n* are evaluated by the intercept and slope of the plot of ln*q*_*e*_ *vs*. ln*C*_*e*_.

To examine sorption isotherms, 0.5 g of adsorbent (BC300, BC600, soil, soil with BC300, and soil with BC600, respectively) was mixed uniformly with 50 mL of solution with different concentrations of Cu(II) (i.e., 10, 50, 100, 300, 500, and 700 mg L^−1^) in a 100 ml centrifuge tubes, and the solution pH was adjusted to 5.0 ± 0.1 by 0.1 mol L^−1^ NaOH or 0.1 mol L^−1^ HCl. Furthermore, the mixture was shaken with a speed of 150 rpm at 25 °C by a thermostatic oscillator (ZC-100B, Shanghai, china). After 25 h, the extract was separated from the adsorbent by a centrifuge at 4000 rpm for 15 min at 25 °C, and the supernatant was filtered immediately through a 0.45 μm microfiltration membrane. The concentration of Cu(II) was determined by AAS at 324.7 nm. The amount of Cu(II) adsorbed on different adsorbents was calculated by Eq. (3)^71^:

3 $${q}_{e}=\frac{({C}_{i}-{C}_{e})\times V}{{\rm{m}}}$$

where *q*_*e*_ (mg g^−1^) is the amount of metal ions adsorbed at the equilibrium time; *C*_*i*_ and *C*_*e*_ (mg L^−1^) are the metal ions concentrations of the initial and equilibrium aqueous phases, respectively. *V* (L) represents the volume of solution, and m (g) is the mass of the adsorbent.

#### Adsorption kinetics experiments

Three kinetics models, the intra-particle diffusion, the pseudo-first-order, and the pseudo-second-order models, were used to investigate the adsorption kinetic behaviors of metal ions on the adsorbent. These three models can be expressed as Eqs (4,5 and 6), respectively^75,84,85^:

4 $${q}_{t}={K}_{p}{{\rm{t}}}^{1/2}+C$$

where *q*_*t*_ (mg g^−1^) is the amounts of metal ions adsorbed at time *t*, *K*_*p*_ (g mg^−1^ h^−1/2^) is the rate constant of intra-particle diffusion obtained from the plot of *q*_*t*_ against *t*^1/2^, and *C* (mg g^−1^) is the intercept reflecting the boundary layer effect.

5 $${\rm{lg}}({q}_{e}-{q}_{t})=\,{\rm{lg}}\,{q}_{e}-\frac{{K}_{{\rm{f}}}t}{2.303}$$

6 $$\frac{{\rm{t}}}{{q}_{t}}=\frac{t}{{q}_{e}}+\frac{1}{{K}_{s}{{q}_{e}}^{2}}$$

where *K*_*f*_ (h^−1^) is the adsorption rate constant of pseudo-first-order obtained from the linear plots of log (*q*_*e*_ − *q*_*t*_) against *t*, *K*_*s*_ (g mg^−1^ h^−1^) is the rate constant of pseudo-second-order obtained from the plot of *t/q*_*t*_ against *t*.

Sorption kinetics of Cu(II) was determined by mixing 50 mL of 300 mg L^−1^ Cu(II) solution with 0.5 g of each adsorbent of BC300, BC600, soil, soil with BC300, and soil with BC600, respectively, in a 100 ml centrifuge tube. The mixture, with four replications, was shaken with a speed of 150 rpm at 25 °C by a thermostatic oscillator. After certain periods of time (1, 5, 10, 15, 25, 35, 50, and 65 h), the extract was separated from adsorbent by a centrifuge at 4000 rpm for 15 min at 25 °C, and the supernatant was filtered immediately through a 0.45 μm microfiltration membrane. The concentration of Cu(II) was determined by AAS. And the amount of Cu(II) adsorbed on different adsorbents was calculated by Eq. (3).

#### Adsorption thermodynamic experiments

To check whether the adsorption process is spontaneous, we calculated the thermodynamic parameters, such as enthalpy (ΔH°), entropy (ΔS°) and Gibb’s free energy (ΔG°) by Eqs (7) and (8)^86,87^.

7 $${\rm{\Delta }}G^\circ =-\,RT\,\mathrm{ln}\,{K}^{\theta }$$

8 $${\rm{\Delta }}G^\circ ={\rm{\Delta }}H^\circ -T{\rm{\Delta }}S^\circ $$

Equation (7) can be written as:

9 $$\mathrm{ln}\,{K}^{\theta }=-\,{\rm{\Delta }}G^\circ /RT=-\,{\rm{\Delta }}H^\circ /RT+{\rm{\Delta }}S^\circ /R$$

where *R* is the gas constant (8.314 J mol^−1^ K^−1^), *T* is the absolute temperature, *K*^*θ*^ is the thermodynamic equilibrium constant calculated by Eq. (10). ΔH° and ΔS° can be obtained from the plot of ln*K*^*θ*^ against *T*, ΔG° can be calculated by Eq. (8).

10 $${K}^{\theta }=\frac{{{\rm{C}}}_{{\rm{i}}}-{{\rm{C}}}_{{\rm{e}}}}{{{\rm{C}}}_{{\rm{e}}}}$$

The adsorption thermodynamic experiments were carried out by adding 0.5 g adsorbent (BC300, BC600, soil, soil with BC300, and soil with BC600, respectively) to 50 mL of 300 mg L^−1^ Cu(II) solutions in a 100 ml centrifuge tube. The mixture was shaken with a speed of 150 rpm at varying temperatures (15 °C, 25 °C, and 35 °C, respectively) for 25 h by a thermostatic oscillator. The extract was separated from the adsorbent by a centrifuge at 4000 rpm for 15 min at 25 °C, and the supernatant was filtered immediately through a 0.45 μm microfiltration membrane. The concentration of Cu(II) was determined by AAS. The amount of Cu(II) adsorbed on different adsorbents was calculated by Eq. (3).

All adsorption experiments were performed in duplicate under identical conditions, and the average values are presented in this study.

## Data Availability

The data has been deposited in figshare and are available at 10.6084/m9.figshare.8320700.
